# 

**Published:** 1884-09

**Authors:** 


### CONTENTS.

ORIGINAL COMMUNICATIONS: PAGE.

I. Chronic Adhesive Perimetritis. By James H. Etheridge, Chicago.... 193

II. A Synopsis of the Medical Botany of Illinois. By J. M. G. Carter, M.A., M.D., PH. B. 214

EDITORIAL:

The Samuel D. Gross Professorship of Pathological Anatomy.... 250

Shall Medical Officers of the Army Engage in Private Practice.... 253

CORRESPONDENCE:

The S. D. Gross Professorship of Pathological Anatomy.... 255

Shall Army Medical Officers Engage in Private Practice.... 2[illegible]9

BOOK REVIEWS: PAGE.

Die Diphtherie.... 263

The Theory and Practice of Medicine.... 263

Post-Nasal Catarrh and Diseases of the Nose Causing Deafness.... 264

Hooper's Physician's Vade Mecum.... 265

SOCIETY PROCEEDINGS:

Chicago Medical Society, May Meeting.... 267

Report of Committee on State Medicine.... 274

Chicago Medical Society, June Meeting.... 279

Median Lithotomy.... 282

MARINE HOSPITAL SERVICE:

Official List of Changes of Stations and Duties of Medical Officers of the United States Marine Hospital Service.... 286

